# Genetic counselling in the era of genomic medicine

**DOI:** 10.1093/bmb/ldy008

**Published:** 2018-04-02

**Authors:** Christine Patch, Anna Middleton

**Affiliations:** 1 Florence Nightingale Faculty of Nursing, Midwifery and Palliative Care, King’s College London, 57 Waterloo Road, London; 2 Genetic Counselling, Genomics England, Queen Mary University of London, Dawson Hall, London; 3 Society and Ethics Research, Connecting Science, Wellcome Genome Campus, Hinxton, UK; 4 Faculty of Education, University of Cambridge, 184 Hills Road, Cambridge, UK

**Keywords:** genetic counselling, genetic counsellors, genomic medicine, policy, practice

## Abstract

**Background:**

Genomic technology can now deliver cost effective, targeted diagnosis and treatment for patients. Genetic counselling is a communication process empowering patients and families to make autonomous decisions and effectively use new genetic information. The skills of genetic counselling and expertise of genetic counsellors are integral to the effective implementation of genomic medicine.

**Sources of data:**

Original papers, reviews, guidelines, policy papers and web-resources.

**Areas of agreement:**

An international consensus on the definition of genetic counselling. Genetic counselling is necessary for implementation of genomic medicine.

**Areas of controversy:**

Models of genetic counselling.

**Growing points:**

Genomic medicine is a growing and strategic priority for many health care systems. Genetic counselling is part of this.

**Areas timely for developing research:**

An evidence base is necessary, incorporating implementation and outcome research, to enable health care systems, practitioners, patients and families to maximize the utility (medically and psychologically) of the new genomic possibilities.

## Introduction

The focus and title of the UK Chief medical officers 2016 report ‘Generation Genome’, emphasizes the importance currently placed on developments in genomic medicine.^1^ Advances in genomic technology will deliver data at affordable scale and has the potential to deliver cost effective targeted diagnosis and treatment.

The drive towards the vision of genomic medicine is informed by various large-scale sequencing projects across the world. One of the UK 100,000 Genome Project’s aims is to transform the National Health Service by integrating genomics into mainstream clinical care.^2^ The opportunities and challenges identified for genomic medicine 10 years after the publication of the first draft of the Human Genome Sequence^3^ have been borne out in the experience of the 100,000 Genomes Project and the other projects internationally. There are many challenges ahead, to name but a few, these include: controversies around data use and sharing across international borders, the need for standardized methods for interpretation of genomic data, the streamlining of reliable quality-assured results delivery within a clinical setting, maximized clinical utility of results while minimizing the uncertainty of the information and managing uncertainty about which data to return in the clinical setting. However, learning from existing clinical services, the UK 100,000 Genomes Project and other similar projects are already enabling genomic diagnoses to be delivered within health care settings.^2,4^

Traditionally, genetic testing and genetic counselling within the UK has taken place within NHS regional genetics centres in tertiary care. The focus has been predominantly on rare syndromes and disorders showing Mendelian inheritance patterns. Genetic counsellors work as part of the multidisciplinary health care team providing expertise both in the science of genetics as well as facilitating the inevitable inter-generational (and often emotional) conversations from the psychological impact of the science. There is an ongoing debate as to the tasks of genetic counselling in this traditional domain and the developing roles in relation to genomic medicine.^5–8^

‘Genetic counsellor’ is an internationally recognized professional title with practitioners having specialist education, assessed competencies in genetics (and now genomics) combined with counselling skills.^6,9,10^ In England, as part of the workforce development initiated through the investment in the development of genomic medicine, the NHS scientist training programme offers a genomic counselling branch to equip practitioners to work under the current professional title of genetic counsellor. This is a new addition to existing routes into practice in the UK which have been provided through master’s level training followed by a competency based assessment and registration with the Genetic Counsellor Registration Board. The ongoing demand for a skilled and competent workforce will require a variety of routes for health care practitioners to develop relevant skills.

It is probable that all health care practitioners will be engaging with genomic medicine in time and as it is ‘mainstreamed’ at scale, health professionals will increasingly be asked to communicate and manage the results from genomic testing. The question is how much of what they will be doing is simply ‘having a conversation based on genomic data’ versus applying some aspects of genetic counselling skills and practice, i.e. will all health professionals be ‘doing’ genetic counselling to some degree? The professional bodies representing genetic counsellors are in a strong position to guide this to resolution. As yet it is unknown what, of genetic counselling skills and theory, will and should be translated into mainstream practice.^5,6^

There is a growing market in genetic tests available directly to the consumer and both the American College of Medical Genetics and the European Society for Human Genetics have recommended that genetic counselling is made available to online customers. This is further discussed in a recent position paper^11^ and will not form the focus of this review.

In this review the developments in and challenges for genetic counselling, both as an activity and a profession, will be discussed in relation to genomic medicine, but focussed on practice within health care settings.

## Sources of data

A structured search of Web of Science, PubMed, Embase and CINAHL was conducted using the search terms ‘genetic counselling’ and ‘genomic medicine’. Articles were included if they presented empiric data or detailed discussion on the practice of genetic counselling in health care settings. They were excluded if they referred solely to healthy population genome sequencing studies, sequencing in a research setting or direct to consumer or commercial genetic testing. Bibliographies were scanned to retrieve additional relevant articles and policy documents. The websites from Professional Societies relevant to genetic counselling practice in Europe, North America and Australasia were reviewed for applicable policy documents. The summaries below offer an overview of the issues represented in published material. However, this is not a systematic review, and the topics selected for presentation are guided by the experience of the two authors. Our aim is to reflect on the climate of genomic medicine, view this through a genetic counsellor lens and articulate the issues of importance to genetic counselling practise.

## Findings

### Genetic counselling

There is consensus among professional bodies around the world that the act of genetic counselling is a client-centred communication process with the aim of helping patients understand, adapt and adjust to the medical or psychosocial consequences of genetic contributions to disease.^12^ Definitions of genetic counselling have evolved over time, however professionals trained in genetic counselling traditionally have assessed an individual’s risk of a genetic disorder, prepared individuals for genetic testing, communicated the results and assisted the management of the patients’ genetic disease as well as preparing and supporting the individual to contact their relatives also at risk of the same disease. Patients would attend genetic services either to obtain a diagnosis of a genetic condition or because they knew of a condition in their family and wanted to understand their own options and choices for managing the consequences of the condition.

There is long history of conceptualizing genetic counselling as teaching and counselling or more recently a very circumscribed form of psychotherapy.^6,13^ In the era of genomic medicine the debate as to where to focus expertise continues.

### Genetic/genomic counselling and genomic medicine

In discussions of the implications of genomic medicine, the term ‘genomic counselling’ has started to be used. It is unclear how this is differentiated from the act of genetic counselling but it is related to the developments in technology and the desire to create genomic medicine defined by the National Human Genome Research Institute as:

*an emerging medical discipline that involves using genomic information about an individual as part of their clinical care (e.g. for diagnostic or therapeutic decision-making) and the health outcomes and policy implications of that clinical use*.

The earliest use of the term ‘genomic medicine’ was in the title of a Commentary paper, in 2009. Here the argument was made that information from a whole genome should be part of an integrated electronic health record. The implication being that genomic data would be available for use across health services in all medical specialities. The whole clinical team would be involved in using this data in the management of patients.^14^ We now know that much of the relevance, across clinical specialities, will be using molecular testing for screening, diagnosis and to guide treatment^15^ and thus genomic medicine will eventually become simply ‘medicine’. The focus on technology and data in definitions of genomic medicine is important but there should also be a recognition of how the information generated is incorporated into the lives, experiences and health care decisions of individuals.

The challenges relating to storing, interpreting and using data generated from genome sequencing are well rehearsed and remain.^16,17^ How to communicate and make decisions about ‘secondary’, ‘incidental’ or ‘additional findings’ are also an active area of discussion.^18^ The terminology around these findings is used inconsistently but broadly they relate to results within the sequence that are found or searched for deliberately, that are secondary to the reason for doing the sequence to start with. There is also a confusion between issues relating to sequencing technologies used in: (a) a clinical setting as a technology to answer a clinical question with return of results related to that clinical question, (b) sequencing used in a healthy population with the intention of returning clinically actionable results and (c) sequencing used for research cohorts with the possibility of returning ‘results’ to participants (including raw sequence data). The response to these challenges will depend on the context within which the technology is being used.

**Fig. 1:**
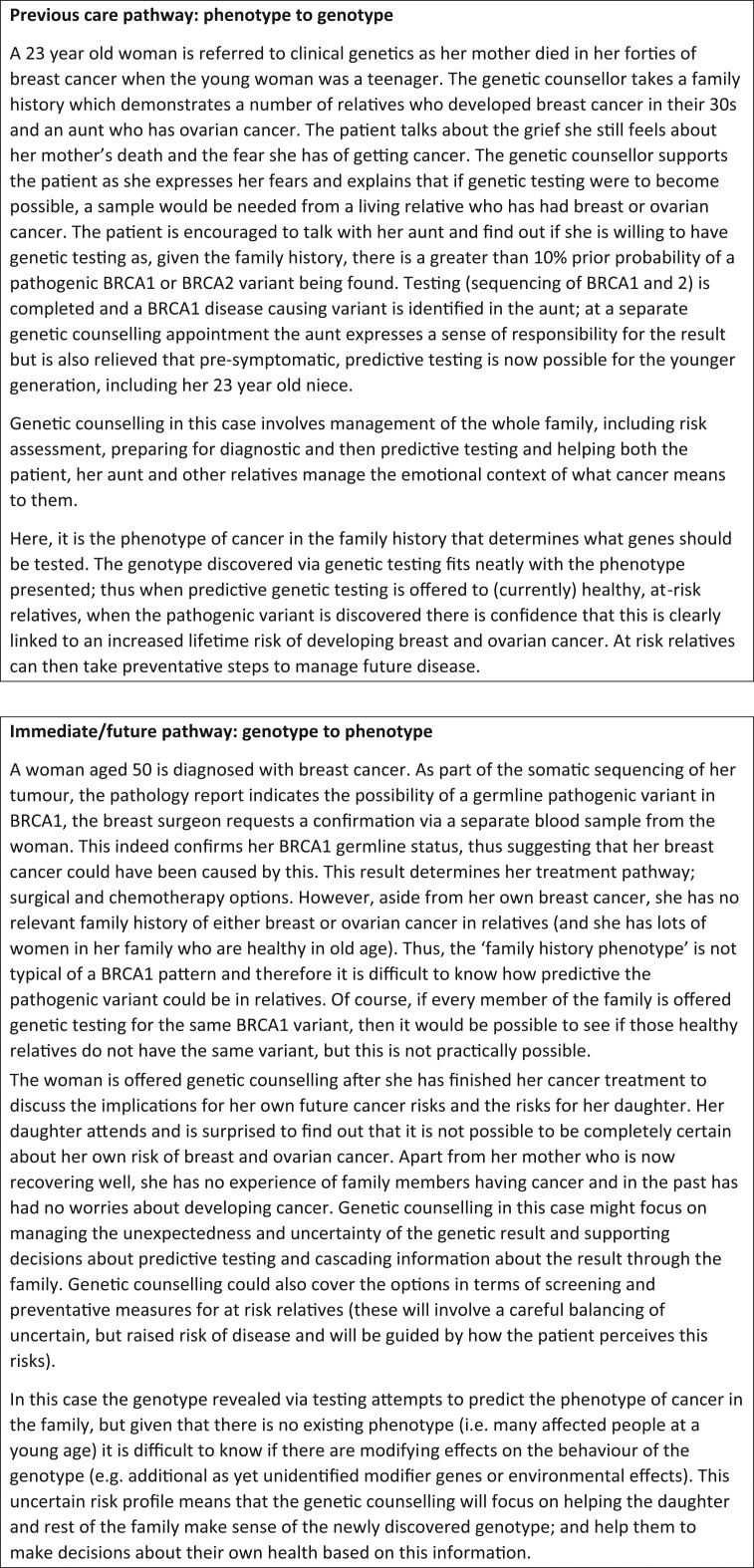
Previous and future care pathways involving genetic counselling.

Genetic counsellors have skills enabling them to be involved in all stages of the patient’s pathway through genomic medicine, however there is uncertainty, as genetic technologies become more routinely used in health care, what aspects of their practice is necessary and at which time points. It is probable that as genomic medicine becomes mainstream, certain roles that are currently considered as an integral part of genetic counselling practice, may become part of other clinicians’ roles (Fig. 1).

### Consent for genomic testing

Historically clinical genetics services were the only healthcare setting where genetic testing could be accessed. Now, genomic technologies can be accessed across the entire healthcare service. Given the extensive experience in working with genetic technologies, clinical genetics services have developed systems for patient management that may be applicable within non-genetics specialisms. One of these systems relates to the process of consent. The ability to offer presymptomatic testing for inherited diseases such as Huntington’s Disease in the 1990s resulted in the recommendation for multi-session counselling prior to taking blood for analysis, thus giving the patient time to psychologically adapt and prepare for a possible future condition where there was no effective treatment or cure.^19^ The two pre-testing counselling sessions focused on information giving about the disease and the genetic risks and exploration of how the patient would incorporate their potential new genetic status into their lives, once they had the results. The aim was to facilitate an informed decision about whether to have the test, who should be tested and when the test should be performed and also to address the emotional context of the test decision. This took into account the presence of a highly penetrant single gene disorder, concerns about the history of genetics and incorporated the values of autonomy and non-directiveness into the genetic counselling approach.

As more presymptomatic/predictive testing has been possible for conditions where prevention or management strategies can be recommended, such as cancer predisposition genes, genetic counselling protocols have been shortened. However there is still an emphasis on provision of information and decision making about the test exploring the emotional and psychosocial implications.^20,21^ As genetic technologies become incorporated into more routine health care decision-making about treatment and management they will become a routine part of diagnostics. It may be that in this context, less focus is placed on the decision about whether to have the test, with a shift to more exploration, post-test as to what the results mean. The consent conversation then moves – where the main body of information about the genetic test result occurs post-test as opposed to pre-test. Once a genetic result is known there becomes a need to have a conversation about the relevance of this to treatment and clinical decision making within the pathway of resolving a medical issue, rather than a personal decision about the desirability and consequences of obtaining genetic information. However, the pre-test information for at-risk relatives, who do not currently have genetic disease, is still as relevant as it ever was and would likely fall within the domain of existing genetic counselling practice. Thus, the care of ‘at risk relatives’, who are not yet unwell, but who are potentially pre-disposed to developing disease, should be referred by primary and secondary care, into tertiary genetics services.

Genetic counsellors have specific training and skills in communication and genetic science but are a scarce resource. There is a tension developing in where to focus this resource and a need for an evidence base to determine what is best practice and what competencies and skills are required for the health professionals who are responsible for the delivery of genomic health care.

The availability of increasingly extensive genetic analyses has raised the possibility of more uncertain findings and findings unrelated to the reason for requesting the test. Concern is expressed as to how to ensure appropriate consent for sequencing analysis in various contexts, how to manage the uncertainty of the interpretation of the data and how to communicate that uncertainty back to patients.^4,22,23^ Questions have arisen as to how information about these possible outcomes should be incorporated into the consent process and how to ensure that consent is appropriate.^24^ In 2013 the American College of Medical Genetics created some controversy by suggesting that all patients receiving genome sequencing should have an analyses performed to identify actionable genetic variants in a specific list of genes linked to serious, life-threatening disease. These guidelines have recently been updated with a process for nominating new genes.^25^ The recommendation is that the patient can choose to opt-out of receiving these secondary findings. Other policy statements concerning the clinical use of whole genome technologies, recommend minimizing the chance of generating secondary findings by performing clinically directed tests.^26^ In the UK 100,000 Genomes Project the decision has been made to limit the possible additional findings that will be analysed.^2^

The complexities of decisions about testing and informational requirements have focused genetic counselling activity at the beginning of the patient pathway through the genetic testing process. The provision of more detailed information and more choices regarding results has been explored in the context of research studies, where results from sequencing have been returned in the healthy populations and in participants recruited from health care services. Various studies have also explored different ways of presenting information and eliciting preferences for the return of additional findings and results.^27–29^ Such research offers helpful exploration for the mechanisms that could be applied to clinical pathways. Recent empirical work interviewing health care professionals consenting patients for genomic testing has reiterated that being informed about all possible outcomes of genetic testing is not possible and moreover may not always reflect appropriate consent.^30^ The time taken for this detailed consenting process has also been identified as a problematic issue.^31^ Given the extensive experience that genetic counsellors have developed over the years in consenting patients for genetic testing, their insight offers a valuable contribution to new practitioners of genomic medicine. The consent conversation for a genomic test is multi-faceted. It should cover: the implications of the result, both for the individual patient and also their family (with a recognition that patients are expected to inform their relatives about their result so that they can seek out their own testing where relevant); the types of result that may come back from the laboratory (i.e. pathogenic, uncertain or benign) and what each of these will mean in terms of future risk and disease management and plans for genomic data storage, protection and sharing.

### New roles for genetic counsellors

As the technologies used for genetic testing have increased in complexity, genetic counsellors have become involved in new roles. The global number of genetic counsellors is estimated at only 7000, practising in at least 28 countries with over 60% in North America. In North America, Canada and South Africa genetic counsellors are increasingly employed by laboratories. A recent survey identified the main roles as customer liaison/case coordination and variant interpretation/result reporting.^32^ A survey in Europe, prior to setting up the genetic counselling European Registration system, did not identify practitioners working is this role yet.^33^ In Europe and the UK, as discussed previously, most genetic counsellors work in multidisciplinary teams within the setting of specialist genetic health care. The teams include medically qualified clinical geneticist and laboratory scientists. Emerging models of genomic medicine emphasize the iterative nature of variant interpretation with input from the bioinformatician, the laboratory scientist and the clinician.^18^ These multidisciplinary teams will often now include a genetic counsellor.^34,35^

Understanding of genome sequencing technologies and variant interpretation is identified as an important part of the knowledge of genetic counsellors and other health professionals. With increasing volumes of sequencing data there are a plethora of bioinformatics tools being developed which curate and synthesize evidence for variant classification in line with professional guidelines.^36^ It is probable that these bioinformatics tools will become more sophisticated over time and remove the need for as much human intervention as there is today.

In addition to having roles within laboratories, as well as multidisciplinary genetics teams, genetic counsellors are now moving further afield. Genetic counsellors, will often practise autonomously within non-genetics specialisms, for example, joining the specialist ophthalmology, cancer, endocrine, ENT or dermatology clinic. With the mainstreaming of genomic medicine they are now being called upon to train and educate their non-genetics colleagues in genomics. Increasingly genetic counsellors also offer their own independent private practice, in the UK this is happening already within the cancer and pre-implantation genetic diagnosis field. They also may practice full time as academics in education or research as well as policy and industry.^5^

Increasingly these roles are being developed independently of the tertiary genetic services in a similar way to the autonomous roles that have developed in other countries for example in North America. As noted above although numbers are small, genetic counsellors practice across both developed and developing countries. How models of care will be implemented is unclear and the mainstreaming conversation includes all members of the health care team. International networks and opportunities for sharing expertise, learning and good practice are growing in genetic counselling, nursing and midwifery and medicine. How this impacts on the development of services in these different health care systems is beyond the scope of this review but is an important question.

The implementation of genomic medicine across the whole healthcare service has the potential to significantly increase the demand for clinically practising genetic counsellors. If, as predicted, hundreds of thousands more patients have a genomic test as part of their diagnostic pathway, any complications from this will result in a post-results referral back into clinical genetics. It will be genetic counsellors who will likely pick up these referrals (having expertise in explaining the significance of genomic results and help the patient make sense of these) and also sort out the cascade family testing. For every new genetic diagnosis made in a non-genetics healthcare setting, the clinician who orders the test and delivers the results, *should* have a conversation about the impact of these for relatives. Given that non-genetics clinicians would not routinely care for at-risk, healthy relatives, the future management of such ‘patients in waiting’ will be enabled by a referral into tertiary genetic counselling services. Workforce planning and the need for more skilled staff is recognized as an issue that needs addressing both in the UK and internationally. The genetic counselling work force is integral to this.

### Giving results and managing the consequences

The large amount of data generated by genomic technologies and the potential for uncertainty is a feature of genetic testing in the genomic era. It has been suggested that genomic medicine will feature heavily on enabling patients to manage and adapt to uncertainty.^22^

In the past, genetic services focussed on rare syndromes and inherited genetic disorders. Families with these types of conditions will still be a significant part of genomic medicine practice. The majority of genomic testing used clinically to date is in the diagnosis of single gene disorders. As discussed previously, definitions of genetic counselling as a client-centred communication process incorporate an aim of adjustment and adaptation to the medical or psychosocial consequences of genetic contributions to disease.^12^ It has been noted however that the ‘teaching model’ within genetic counselling practice, has become predominant with less time being given to eliciting client concerns or providing psychological care.^37^ There is a debate in the literature as to whether the psychotherapeutic aspect of managing genetic information should be made more prominent^13^ partly in response to acknowledging that all health care professionals in the future will be having conversations with their patients about genomic results. Such conversation, whilst it might draw on good medical communication skills, is *not* genetic counselling and while health care professionals may need support in delivering the genomic information they will not be providing genetic counselling per se. Genetic counselling attends to both the educational component and the psychosocial and emotional component of the genomic information and incorporates discussion with relatives.^8^

There is evidence that genetic counselling can lead to a range of positive outcomes including increased knowledge, perceived personal control; positive risk behaviours and improved risk perception.^38^ Genetic counsellors have been engaged in research to develop their practice and interventions to assist patients and their families in managing the genetic information.^39,40^ However it is recognized that there is a need for greater evidence base for genetic counselling in order that services may continue to evolve to meet the changing needs of patients and families with genetic conditions.^10^

In the same way that developing roles in laboratories and variant interpretation are amenable to technology solutions that assist in test selection or interpretation of variants, the educational and information provision aspect of genetic counselling may also lend itself to innovative ways of delivery. For example in August 2017 a genetic testing company announced the formation of a start-up company to develop an internet algorithm (chatbot) to guide patients through their genetic tests. However the provision of information about tests and their potential results cannot ever replace the psychosocial conversation, based on a two-way, patient-led conversation that addresses the needs of patients to adapt and adjust and incorporate this new knowledge into their lives.

## Genetic counselling in the era of genomic medicine

Examination of the literature and discussions with experts has demonstrated that genetic counsellors are involved at all stages in the pathway of the patient journey through genomic medicine. At the beginning, with genetic risk estimation, decision-making about testing and conversations about consent; in the management and interpretation of results; at the point at which the result enters into the health care system and is returned to the clinician and the patient, at the follow up of patients in mainstream and in helping the patient to seek out and communicate the results within their family. Genetic counsellors are also involved in supporting the patient to adapt to the genetic information and manage the psychosocial consequences of this as it is relevant to the wider family and future generations.

As genomic medicine develops it will become clearer as to what aspects of genetic counselling will become part of mainstreamed health services and what aspects will remain within the domain of the specialist genetics workforce. The current focus on information provision prior to the test and managing information generated by the genomic analysis may change as solutions are developed that make this more routine. However, the consequences of the genetic information, particularly as it relates to conditions with implications for future health and familial implications, may need more specialist genetic counselling skills in order that patients and families can adjust and adapt to the information and use it effectively to maximize the health gains from genomic medicine and minimize potential harms.

In order to increase the evidence base for practice there is an urgent need for funding and research resources to be focussed on implementation and outcome research. This needs to explore how to enable health care systems, practitioners, patients and families to maximize the utility of the new knowledge. Genetic counsellors with their experience of implementing genetic medicine should be at the heart of effective and responsible implementation of genomic medicine.
